# Clinical characteristics of hospital-onset *Pneumocystis* pneumonia and genotypes of *Pneumocystis jirovecii* in a single tertiary centre in Korea

**DOI:** 10.1186/s12879-015-0847-6

**Published:** 2015-02-26

**Authors:** Tark Kim, Sang-Oh Lee, Hyo-Lim Hong, Ju Young Lee, Sung-Han Kim, Sang-Ho Choi, Mi-Na Kim, Yang Soo Kim, Jun Hee Woo, Heungsup Sung

**Affiliations:** Department of Internal Medicine, Soonchunhyang University Bucheon Hospital 170 Jomaru-ro, Bucheon-si, Gyeonggi-do 420-767 Republic of Korea; Department of Infectious Diseases, Asan Medical Center, University of Ulsan College of Medicine, 88, Olympic-ro 43-gil, Songpa-gu, Seoul 138-736 Republic of Korea; Department of Laboratory Medicine, Asan Medical Center, University of Ulsan College of Medicine, 88, Olympic-ro 43-gil, Songpa-gu, Seoul 138-736 Republic of Korea

**Keywords:** *Pneumocystis* pneumonia, Large ribosomal subunit of mitochondrial rRNA, Transmission, Epidemiology

## Abstract

**Background:**

*Pneumocystis* pneumonia (PCP) may develop as a clinical manifestation of nosocomial pneumonia by means of either reactivation of resident *P. jirovecii* or *de novo* infection. However, there have been no studies describing the clinical characteristics of hospital-onset PCP.

**Methods:**

A retrospective review of medical records was performed to identify episodes of hospital-onset PCP in a tertiary care centre in Korea between May 2007 and January 2013. We investigated whether human-to-human contact during hospitalisation contributed to PCP development by molecular analysis of the genes encoding mitochondrial large ribosomal subunit (*mtLSU*) rRNA and dihydropteroate synthase (*DHPS*) and a review of hospitalisation history.

**Results:**

During the study period, 129 patients (130 episodes) were diagnosed with PCP. Of these, respiratory specimens from 94 patients during 95 PCP episodes were available for analysis. Sixteen episodes (16.8%) were categorised as hospital-onset PCP. There was a trend toward a higher proportion of haematological malignancy (43.8% [7/16] *vs*. 20.3% [16/79]; *P* = 0.058) in patients with hospital-onset PCP compared to patients with community-onset PCP. *mtLSU* genotype 1 was the most common, occurring in 41 (43.2%) patients. There were four possible cases of nosocomial transmission. Mutation in *DHPS* was not observed in any PCP episode.

**Conclusions:**

PCP can be one of the causes of nosocomial pneumonia, although the mode of acquisition and transmission of *P. jirovecii* remains uncertain. *mtLSU* genotype 1 is the predominant *P. jirovecii* strain in Korea.

## Background

*Pneumocystis* pneumonia (PCP) is a major opportunistic infection in immunocompromised patients principally acquired and transmitted via an airborne route [1]. It has been proposed previously that *Pneumocystis jirovecii* infects the host during childhood, becomes part of the resident microbial flora and remains latent for extended periods, reactivating when the host becomes immunocompromised. This theory is based on the observation that most humans become seropositive for *P. jirovecii* early in life [2], and that the colonisation of *P. jirovecii* in animal models persists over long periods of time [3]. By contrast, other researchers suggest that *P. jirovecii* infects transiently and that active transmission is possible. This idea is supported by early clearance of *P. jirovecii* from the lungs of adult animals [4] and detection of mutations in dihydropteroate synthase (*DHPS*) in human immunodeficiency virus (HIV)-infected patients without prior sulfamethoxazole prophylaxis [5]. Recent reports of clusters or outbreaks of a single genetic strain of *P. jirovecii* also suggest *de novo* infection by human-to-human transmission [6-10].

Based on these previous observations, PCP may develop as a clinical manifestation of nosocomial pneumonia by means of either reactivation of resident *P. jirovecii* or *de novo* infection [11]. Damiani *et al.* provided additional data supporting *P. jirovecii* exhalation by infected patients, showing that a full match of *P. jirovecii* genotypes was found for 4 (57.1%) pairs of pulmonary and room air samples [7]. Therefore, *de novo* infection by human-to-human transmission may be a cause of some of PCP development during hospitalisation. Thus, it is necessary to apply measures preventing airborne transmission of *P. jirovecii* in hospitals.

In this study, we retrospectively identified the episodes of hospital-onset PCP. We performed a molecular analysis of the genes encoding mitochondrial large ribosomal subunit (*mtLSU*) rRNA and *DHPS* and a review of hospitalisation history of patients with PCP to investigate whether human-to-human contact during hospitalisation contributed to PCP development.

## Methods

### Ethics statement

This work was approved by the Research Ethics Committee at Asan Medical Centre, Seoul, Republic of Korea. Informed consent was waived by the Institutional Review Board of Asan Medical Center since this work was a retrospective study without intervention and did not involve extra clinical specimens.

### Study design and patients

This study was performed at Asan Medical Center, a 2,700-bed tertiary care teaching hospital, from May 1, 2007 to January 31, 2013. The study included patients with compatible symptoms and radiological findings confirmed as PCP by a direct immunofluorescence assay (Light Diagnostics™ *Pneumocystis carinii* DFA Kit, Millipore, Billerica, MA, USA) using respiratory specimens. We excluded patients whose respiratory specimens were not available for molecular analysis. Respiratory specimens were not collected between September 1, 2009 and September 31, 2011 because the principal investigator was on sabbatical leave. During a retrospective medical review, we identified episodes of hospital-onset PCP which was defined as pneumonia arising more than 5 days after admission when no signs and symptoms compatible with PCP were documented at the time of admission. The 5 day-cutoff for diagnosing hospital-onset PCP is same as that used in previous studies of late-onset ventilator-associated pneumonia [12]. Other patients were considered to have community-onset PCP.

### Data collection

The patients’ medical records were reviewed retrospectively, and clinical information was collected, including demographics, underlying diseases and conditions, hospitalisation history, reason for admission, signs and symptoms of PCP, radiological findings, history of prior prophylaxis against PCP, laboratory findings (neutrophil and lymphocyte counts in bronchoalveolar lavage [BAL] fluid, absolute neutrophil and lymphocyte counts, lactate dehydrogenase, and C-reactive protein), initial severity of PCP, treatment regimens and response, need for mechanical ventilation and 30-day mortality from the time of initial PCP diagnosis. Using this information, we compared the clinical characteristics of patients with hospital-onset and community-onset PCP. A possible case of nosocomial transmission was defined as a patient with a history of admission to the same ward in which another patient infected with a genetically identical strain of *P. jirovecii*, determined by *mtLSU* and *DHPS* sequencing, had stayed.

### Genotyping

The majority of specimens (n = 92) were acquired through from BAL obtained using a fibre-optic bronchoscope and standard techniques. Three specimens obtained by endotracheal aspiration. We performed a retrospective molecular analysis of the *P. jirovecii mtLSU* and *DHPS* loci. BAL specimens (350 μl) were treated with proteinase K and DNA was extracted using QIAamp DNA Stool Mini Kits (Qiagen, Valencia, CA, USA) according to the manufacturer’s instructions. The single-copy *DHPS* gene was amplified by nested PCR using the primers F1 and B45 (first round) and AHUM and BN (second round) in all positive samples, as described previously [13,14]. *mtLSU* rRNA gene was amplified by nested PCR using the primers pAZ102-E and pAZ102-H (first round) and pAZ102-X and pAZ102-Y (second round) in all samples, as described previously [2,15]. Amplicons were purified using a Power Gel Extraction kit (TaKaRa Bio Inc., Shiga, Japan) and directly sequenced on an ABI Prism 3130*xl* genetic analyser (Applied Biosystems, Foster City, CA, USA) using a BigDye Terminator v. 3.1 cycle sequencing kit (Applied Biosystems). *DHPS* genotypes were as follows: genotype 1, 165A and 171C (resulting in Thr and Pro); genotype 2, 165G and 171C (resulting in Ala and Pro); genotype 3, 165A and 171T (resulting in Thr and Ser); and genotype 4, 165G and 171T (resulting in Ala and Ser). *mtLSU* genotypes were as follows: genotype 1 = 85C/248C, 2 = 85A/248C, 3 = 85T/248C and 4 = 85C/248T [16].

### Statistical analyses

Chi-square or Fisher’s exact test was used to compare categorical variables, and either Student’s *t*-test or the Mann–Whitney U-test was used to compare continuous variables, as appropriate. Data were analysed using SPSS for Windows, version 15.0 (SPSS Inc., Chicago, IL, USA). A value of *P* <0.05 was taken to indicate statistical significance.

## Results

### Identification of hospital-onset PCP

During the study period, a total of 130 PCP episodes were documented in 129 patients. There were 95 episodes between May 2007 and August 2009, 54 during the interruption period, and 35 between October 2011 and January 2013. Of these, adequate specimens for the study were available for 94 patients experiencing 95 episodes: 70 (73.7%; out of 95 episodes) obtained between May 2007 and August 2009, and 25 (71.4%; out of 35 episodes) obtained between October 2011 and January 2013. In one patient with recurrent episodes, PCP developed in both June 2008 and April 2009. Sixteen patients were categorised as having hospital-onset PCP (Table 1). At the time of admission, none of the patients had signs or symptoms compatible with PCP, and all were admitted as a result of problems not associated with the respiratory system. The median number of days from admission to diagnosis of PCP was 38 (range, 18–63 days).

**Table 1:** **Characteristics of patients with hospital-onset and community-onset PCP**

**Characteristics**	**Hospital-onset PCP** ^*^	**Community-onset PCP**	***P***	**Characteristics**	**Hospital-onset PCP** ^*^	**Community-onset PCP**	***P***
	**(n = 16)**	**(n = 79)**			**(n = 16)**	**(n = 79)**	
Gender, male	9 (56.3)	51 (64.6)	0.58	Interstitial lung disease	0 (0.0)	8 (10.1)	0.34
Age, median years (IQR)	42 (27–59)	52 (39 to 63)	0.13	Connective tissue disease	1 (6.3)	4 (5.1)	1.00
Genotype				Others^†^	2 (12.5)	5 (6.3)	0.34
1	8 (46.7)	33 (41.7)	0.58	History of priory exposure to SMX more than 3 months	2 (12.5)	23 (29.1)	0.22
2	2 (12.5)	3 (3.8)	0.20	Days of BAL from admission, median (IQR)	38 (18 to 63)	2 (1 to 5)	<0.001
3	2 (12.5)	19 (24.1)	0.51	Days of BAL from last chemotherapy, median (IQR)^‡^	17 (9 to 31)	19 (15 to 30)	0.44
4	0 (0.0)	1 (1.3)	1.00	BAL neutrophil, median cells/mm^3^ (IQR)	11 (0 to 80)	19 (7 to 62)	0.43
Mixed	4 (25.0)	23 (29.1)	1.00	BAL lymphocyte, median cells/mm^3^ (IQR)	50 (10 to 177)	48 (12 to 145)	0.84
1 and 2	1 (6.3)	12 (15.2)	0.69	ANC, median cells/mm^3^ (IQR)	5053 (883 to 8863)	5968 (3818 to 9007)	0.18
1 and 3	2 (12.5)	9 (11.4)	1.00	ALC, median cells/mm^3^ (IQR)	416 (130 to 1000)	648 (331 to 1108)	0.11
2 and 3	1 (6.3)	2 (2.5)	0.43	LDH, median IU/L (IQR)	364 (240 to 632)	448 (330 to 620)	0.35
Underlying conditions				CRP, median mg/dL (IQR)	10.2 (4.5 to 14.2)	9.0 (4.0 to 20.3)	0.43
HIV infection	0 (0.0)	13 (16.5)	0.12	Initial severity at diagnosis^§^			
Transplantation				Severe	11 (68.8)	64 (81.0)	0.32
HSCT	3 (18.8)	6 (7.6)	0.17	Treatment			
SOT	3 (18.8)	19 (23.1)	0.76	TMP/SMX usage as initial treatment	14 (93.8)	79 (100)	0.17
Malignancy				Treatment failure to initial regimen^¶^	6 (37.5)	23 (29.1)	0.57
Solid tumour	0 (0.0)	8 (10.1)	0.34	Mechanical ventilation	11 (68.8)	42 (53.2)	0.28
Haematologic	7 (43.8)	16 (20.3)	0.06	30-day mortality	7 (43.8)	18 (22.8)	0.12

Data are numbers (%) of patients, unless otherwise indicated.

^*^Hospital-onset PCP was defined as pneumonia arising more than 5 days after admission when no signs and symptoms compatible with PCP were documented at the time of admission. Other patients were considered to have community-onset PCP.

^†^Autoimmune haemolytic anaemia and Steven-Johnson’s syndrome in patients with hospital-onset PCP. Henoch-Schönlein purpura, severe combined immunodeficiency, idiopathic thrombocytopenic purpura, ulcerative colitis, and unspecified glomerulonephritis in patients with community-onset PCP.

^‡^It was checked only in patients with a haematologic malignancy on chemotherapy.

^§^Severe PCP was defined as partial arterial oxygen pressure <60 mmHg while breathing room air or an alveolar-arterial oxygen difference ≥45.

^¶^Treatment failure was defined as one of the following situations: (1) progressive clinical deterioration as demonstrated by the inability to maintain a stable partial pressure of arterial oxygen despite an increase in the fraction of inspired oxygen, or (2) progressive deterioration of vital signs with a requirement for an increased fraction of inspired oxygen after 7 days of therapy.

ALC, absolute lymphocyte count; ANC, absolute neutrophil count; BAL, bronchoalveolar lavage; CRP, C-reactive protein; HSCT, haematopoietic stem cell transplantation; IQR, interquartile range; LDH, lactate dehydrogenase; PCP, *Pneumocystis jirovecii* pneumonia; SOT, solid organ transplantation; TMP/SMX, trimethoprim/sulfamethoxazole.

### Clinical characteristics of patients with hospital-onset community-onset PCP

Malignancy and transplantation were common underlying diseases and conditions. All patients with connective tissue disease or interstitial lung disease were on immunosuppression. Trimethoprim/sulfamethoxazole was chosen as the initial regimen in almost all patients. Among these patients, 30.5% (29/95) failed to respond to the initial therapy. The 30-day all-cause mortality rate was 26.3% (25/95). A comparison of the clinical characteristics of patients with hospital-onset and community-onset PCP demonstrated no statistically significant differences (Table 1) except a trend toward a higher proportion of haematological malignancy as an underlying disease (hospital-onset PCP, 43.8% [7/16] *vs*. community-onset, 20.3% [16/79]; *P* = 0.058) and a higher 30-day mortality in patients with PCP developing during hospitalisation (hospital-onset PCP, 43.8% [7/16] *vs*. community-onset, 22.8% [18/79]; *P* = 0.12). To adjust for a possible confounding effect on outcome, we performed a subgroup analysis of patients with haematological malignancy. There was no statistically significant difference between the groups, possibly due to the small number of patients (hospital-onset PCP, 3/7 [42.9%] *vs*. community-onset, 2/16 [12.5%]; *P* = 0.14).

### Possible nosocomial transmission of *P. jirovecii*

**Figure 1:**
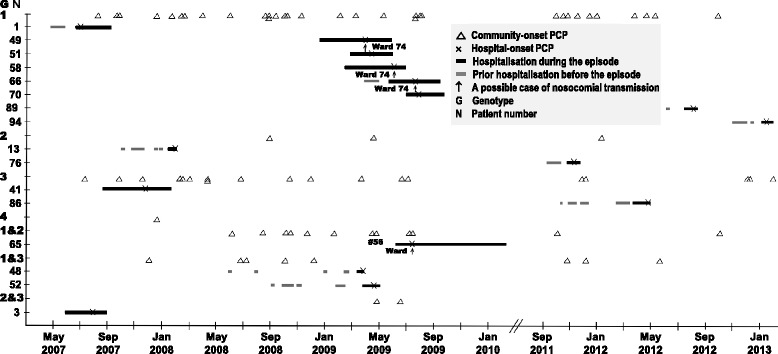
**A transmission map for patients with** ***Pneumocystis*** **pneumonia (PCP) based on mitochondrial large ribosomal subunit (** ***mtLSU*** **) rRNA genotypes.** Genotypes were determined by direct sequencing of nucleotides 85 and 248: genotype 1 = 85C/248C, 2 = 85A/248C, 3 = 85T/248C, and 4 = 85C/248T. Patients 49, 58, and 66 with genotype 1 stayed in ward 74 during a similar period, although they did not share the same room. Patient 65, who developed hospital-onset PCP with a mixed *mtLSU* genotype 1, 2 , stayed in ward 174, in which patient 56 who had a strain with the same mixed genotype was hospitalised. Patients 56 and 65 also did not share the same room

**Figure 2:**
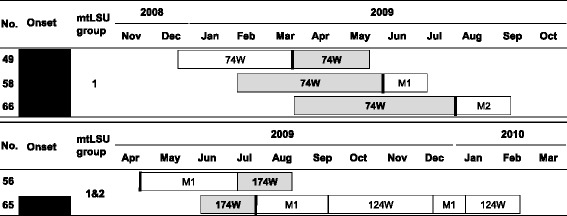
**A transmission map for possible cases of nosocomial transmission of** ***Pneumocystis jirovecii.*** Genotypes of the mitochondrial large ribosomal subunit (*mtLSU*) were determined by direct sequencing of nucleotides 85 and 248: genotype 1 = 85C/248C, 2 = 85A/248C, 3 = 85T/248C, and 4 = 85C/248T. A dark grey box indicates the period of hospitalisation during the PCP episode. The thick vertical line is the time when hospital-onset PCP was diagnosed. Patients 49, 58, and 66 stayed in ward 74 during a similar period. Patients 56 and 65 stayed in ward 174 during the same period. No., case number; Hospital-onset PCP marked by a black box; M1, the first medical intensive care unit; M2, second medical intensive care unit; W, ward

We identified four possible cases of nosocomial transmission (Figure 1). Of these, three patients with hospital-onset PCP with *mtLSU* genotype 1 strains (patients 49, 58, and 66) stayed in ward 74 during a similar period, although they did not share the same room (Figure 2). It was unclear who might be an index patient among them. Patient 65, who developed hospital-onset PCP with a mixed *mtLSU* genotype 1, 2 , stayed in ward 174, where patient 56 who had a strain with the same mixed genotype was hospitalised. Patients 56 and 65 also did not share the same room (Figure 2).

### Genotype distribution of *P. jirovecii*

Genotype results of strains from all episodes, analysed by *mtLSU* and *DHPS* sequencing, are summarised in Table 2. *mtLSU* genotype 1 was the most common, occurring in 41 (43.2%) cases, followed by type 3 in 21 (22.1%) cases. Only one episode with genotype 4 was identified. Strains with mixed genotypes were found in 27 episodes (28.4%). In the patient with recurrent episodes, a mixture of genotypes 2 and 3 was identified in both episodes. *DHPS* mutation was not observed in any episode. Nine of thirteen episodes in HIV-infected patients were with genotype 1, which was a higher proportion than in non-HIV-infected patients (HIV, 69.2% [9/13] *vs*. non-HIV, 37.8% [31/82]; *P* = 0.04).

**Table 2:** **Variant** ***mtLSU*** **and dihydropteroate synthetase (** ***DHPS*** **) genotypes in patients with** ***P. jirovecii*** **pneumonia**

**Genotype (n = 95)**	***mtLSU***		***DHPS***	
	**Nucleotide position/identity**	**No. (%)**	**Nucleotide position/identity**	**No. (%)**
1	85C/248C	41 (43.2)	165 (55)/A (Thr); 171 (57)/C (Pro)	95 (100)
2	85A/248C	5 (5.2)	165 (55)/G (Ala); 171 (57)/C (Pro)	0 (0.0)
3	85T/248C	21 (22.1)	165 (55)/A (Thr); 171 (57)/T (Ser)	0 (0.0)
4	85C/248T	1 (1.0)	165 (55)/G (Ala); 171 (57)/T (Ser)	0 (0.0)
Mixed (total)		27 (28.1)		
1 and 2		13 (13.5)		
1 and 3		11 (11.5)		
2 and 3		3 (3.1)		

## Discussion

In the present study, a significant proportion of PCP cases developed as a clinical manifestation of nosocomial pneumonia. We found a trend toward a higher proportion of haematological malignancy in patients with hospital-onset PCP, which can be explained by the long hospitalisation period during chemotherapy. This finding indicates that PCP should be considered in the differential diagnosis of nosocomial pneumonia and that the list of differential diagnosises was heavily dependent on the immune status and clinical characteristics. *mtLSU* genotype 1 was the most common *P. jirovecii* strain in Korea. We did not identify an association of any particular genetic strain with hospital-onset PCP. Thus, the impact of genotype on the occurrence of hospital-onset PCP and clinical outcomes should be further investigated.

Several studies have suggested that PCP results from *de novo* infection by nosocomial transmission, rather than from reactivation of latent infection. Clustering or outbreak strains with the same genotypes determined by various molecular methods suggest that *P. jirovecii* can be acquired by human-to-human transmission [6-10,17-19]. Additionally, recent studies suggest that the hospital environment and patients colonised with *P. jirovecii* can be a source of transmission [7,20]. Unlike these previous studies investigating whether nosocomial transmission contributed to PCP development during outbreak or clusters, the present study investigated the possibility of nosocomial transmission during the period without outbreak or clusters. Our findings also suggest human-to-human transmission to be a possible mode of *P. jirovecii* acquisition in hospitalised patients. Based on these results, patients with PCP may need to be isolated to prevent human-to-human transmission, and universal surveillance for PCP colonisation may be necessary before admission to wards containing immunocompromised patients. However, these results should be interpreted with caution before they are applied to a strategy for PCP prevention. First, in the present study, most of the patients with hospital-onset PCP had no history of possible nosocomial transmission. This suggests that the *de novo* acquisition of *P. jirovecii*, at least, may rarely occur during hospitalisation. Second, no *DHPS* mutations were found despite a prior history of exposure to sulfamethoxazole in many patients. In a previous study, *DHPS* mutations were not detected in patients in whom PCP developed prior to the widespread use of sulfamethoxazole to treat and prevent the disease [5]. This implies that patients with hospital-onset PCP may be infected from reservoir populations in the community that have not been exposed to sulfamethoxazole. Additionally, the genotype distributions were not different between patients with hospital-onset and community-onset PCP developing. Finally, owing to the long incubation period of *P. jirovecii* [21], it was impossible to strictly differentiate between *de novo* acquisition and reactivation. Therefore, the mode of acquisition and transmission of *P. jirovecii* causing pneumonia during hospitalisation could not be determined in the present study.

The genetic epidemiological features identified in this study determined that the *mtLSU* genotype 1 was the predominant *P. jirovecii* strain, there was a high prevalence of mixed-genotype strains, and *DHPS* mutations were absent. In previous studies in Spain and Japan, genotype 1 was also the most common, occurring in 30% and 49% of patients, respectively, which is comparable to the present study [16,22]. By contrast, genotypes 2 and 3 were reported to be the most prevalent strains in India [23] and Tunisia [24], respectively. In addition, a significant prevalence of mutations in *DHPS* (20–37%) was reported in several countries [16,23-25], while only one *DHPS* mutant was found among 52 strains in Japan [22]. These variable genetic epidemiological findings may be due to geographical differences. We found that genotype 1 occurred more frequently in HIV-infected patients compared to non-HIV-infected patients, which is similar to the report by Montes-Cano *et al.* [16].

The 30-day all-cause mortality rate was 26.3%. This high rate seems to be associated with the high proportion of patients with a non-haematologic malignancy and interstitial lung disease [26]. The rate of treatment failure in the initial therapy was similar to that in our previous study [27]. Our group has already published a number of articles on various clinical issues related to PCP, including the efficacy of salvage therapy regimens and the role of adjunctive corticosteroids [27,28]. Therefore, clinical issues were not extensively reviewed in the present study.

The present study had several limitations. First, because the incubation period of *P. jirovecii* is quite broad (range, 7–188 days) [21], 5 days may not be an appropriate cut-off for differentiating between cases of hospital-acquired and community-acquired PCP. Therefore, we have applied the terms ‘hospital-onset’ and ‘community-onset’ rather than ‘hospital-acquired’ and ‘community-acquired’ PCP. Second, a significant number of patients with PCP (36.9%, 35/130) were not included in the molecular analysis due to a lack of suitable specimens. Third, *DHPS* and *mtLSU* genotyping may not be optimal for epidemiological investigations. To get better discriminatory power, other molecular methods such as multilocus sequence typing should be done [29]. Fourth, other sources of nosocomial transmission, such as patient-to-patient transmission in other hospital departments and the acquisition of *P. jirovecii* from other hospital environments, were not investigated. Moreover, we did not search for *P. jirovecii*-infected individuals without definite signs and symptoms, who can act as problematic reservoirs. Finally, generalisations cannot be made from this study, as it was conducted in a single centre. Different conclusions may result from studies of other populations.

## Conclusions

In conclusion, some episodes of PCP develop as nosocomial pneumonia. PCP should be considered as one of the causes of nosocomial pneumonia in immunocompromised patients, such as those with haematological malignancy. Molecular analysis of *mtLSU* rRNA indicated that a strain of genotype 1 was predominant in Korea. Further investigation should be performed to identify the mode of acquisition and transmission of *P. jirovecii* causing pneumonia during hospitalisation.

## Abbreviations

PCP: *Pneumocystis* pneumonia
mtLSU: mitochondrial large ribosomal subunit
DHPS: Dihydropteroate synthase
BAL: Bronchoalveolar lavage
